# Content analysis of public opinion on sexual expression and dementia: Implications for nursing home policy development

**DOI:** 10.1111/hex.12509

**Published:** 2016-10-05

**Authors:** Maggie L. Syme, Erin Yelland, Laci Cornelison, Judith L. Poey, Ryan Krajicek, Gayle Doll

**Affiliations:** ^1^ Center on Aging Kansas State University Manhattan KS USA; ^2^ School of Family Studies and Human Services Kansas State University Manhattan KS USA

**Keywords:** health‐care policy, nursing homes, public opinion, sexual expression

## Abstract

**Purpose:**

We examined public opinion of sexual expression and dementia to inform nursing home policy and practice.

**Design and Methods:**

A content analysis was conducted on public comments (N=1194) posted in response to a *New York Times* article about a highly publicized legal case involving a husband engaging in sexual acts with his wife who had dementia, living in a nursing home. Researchers utilized constant comparative analysis to code the comments; reliability analysis showed moderately strong agreement at the subcategory level. Data were also coded to indicate whether the commenter thought the couple should or should not have been allowed to be sexual.

**Results:**

One primary theme was identified: conditions necessary for someone to be sexual. Six categories were identified within this theme, with the public commentary considering factors such as marital relationships, intimacy needs and several sexual consent‐related issues as key conditions necessary to be sexual in a nursing home setting. Overall, the majority of commenters were in support of sexual expression for an individual with dementia in the described situation.

**Discussion:**

This study revealed sexual expression among individuals with dementia is a contentious issue with strong public opinions about how this should be managed in a nursing home setting. These opinions should be considered as policy related to sexual expression in nursing homes is developed.

## 1 Introduction

Sexuality is seldom considered in nursing home environments, with few policies or trainings in place to address sexual situations when they arise.^1^ The lack of adequate policy and procedures was exemplified in the legal case of Henry Rayhons, who was accused of sexually abusing his wife, Donna, who had dementia and was living in a nursing home. The public trial raised outrage on both sides—from those that claimed it was a human right for Henry and his wife to be able to express their love for each other to those who believed that he was taking advantage of a person without capacity to consent. Mr. Rayhons was exonerated of sexual assault in the third degree in April of 2015, and it is unknown whether the jury vindicated him based on his wife's perceived ability to consent or the lack of evidence to suggest that sexual intercourse occurred.^2^ Regardless, the actions taken by nursing home staff and law enforcement prior to the trial were noted to be poorly orchestrated and resulted in emotional trauma for Mr. Rayhons. The case, and subsequent public commentary, was reflective of the struggle the public and health‐care systems have with sexual expression and dementia.

The Rayhons were failed by the lack of health‐care policy about sexual expression in long‐term care (LTC). Unfortunately, sexual expression—behaviours ranging from intercourse to intimate behaviours (eg handholding, kissing)—remains largely ignored in nursing homes.^3^ This is due to barriers such as lack of knowledge, more restrictive/negative attitudes about older adult sexuality, and lack of resources to develop policy and train staff.^4^, ^5^ However, experts highlight the need for proactive sexual expression policies for nursing homes that take a resident‐centred approach, in lieu of a more institutional approach where administrators and families are the only voice heard.^6^, ^7^ Discussions that arise from cases such as the Rayhons can and should be used to inform development of resident‐centred health‐care policies regarding sexual expression in nursing homes.

### 1.1 Background

#### 1.1.1 Sex, dementia and long‐term care

Contrary to societal beliefs, sexual and intimate behaviours are exhibited by older adults in nursing home settings^8^, ^9^ and by those that have dementia‐related disease.^10^, ^11^ Behaviours observed in nursing homes include handholding, touching, kissing, intimate relationships, masturbation and—at times—intercourse‐related behaviours.^3^, ^7^

While desire and interest in sex are part of the human experience, the right and need to engage in healthy, intimate or sexual relationships are not often actualized in nursing home settings. This is partially due to the loss of autonomy experienced within a nursing home,^1^ issues with the built environment (eg lack of privacy),^4^ and generally negative/suppressive attitudes towards resident sexual expression from both staff and families.^12^, ^13^

An additional challenge to supporting sexual expression in LTC concerns cognitively impaired residents who may lack sexual consent capacity, or the ability to make ones' own sexual decisions.^7^ Thus, while the desire for and interest in sexual activity may remain, the ability to give consent may be questionable, given his/her level of cognitive impairment. These issues complicate an already difficult task of recognizing sexual rights and needs of nursing home residents.^14^ Thus, homes often err on the side of safety, prohibiting intimacy while leaving the resident with little to no autonomy.

#### 1.1.2 Policy regarding sexual expression in nursing homes

Policies on sexual expression, whether explicitly stated or informally held, have a direct effect on current and future nursing home residents, as they set forth the “rules” by which sexual decisions are made in the facility. Unfortunately, there is a lack of explicit institutional and legal guidance on how to respond to sexual and intimate expression in a way that both supports and protects nursing home residents and staff.^14^, ^15^ A 2015 study of 366 Directors of Nursing (DONs) found the majority of facilities (63.4%) do not have policies addressing any aspect of resident sexuality. Of the facilities that had policies, only 58.6% had written policies with 11.2% of those requiring a physician's order to allow sexual activity.^1^ Lack of policy is also reflected in very few homes being engaged in sexuality training (13.4% of facilities) and staff discomfort towards addressing sexual issues (73.8% report discomfort).^15^

Without adequate policy, a resident's voice may be lost in decision making—such as in the case of the Rayhons—as family and staff are often perceived to hold the right to make such decisions, and their motives may or may not be in the resident's interest.^3^ Further, in the absence of well‐developed policy, administrators in nursing homes may pose certain limits based on factors such as the perceived appropriateness of the acts (eg only permitting sex within marital relationships),^6^ and/or the presence or lack of a dementia diagnosis. Also, facilities may operate by restricting sexual and intimate expression among residents who have been globally deemed to lack consent (eg threshold score on cognitive screener), instead of employing a more valid, nuanced assessment of sexual consent.^7^, ^13^, ^16^ Experts have also called for more nuanced sexual expression policies, which employ a risk continuum of sexual acts and a more complex view of sexual consent.^7^, ^16^

Although experts have posed various recommendations for sexual expression management,^3^, ^6^, ^7^ there is substantial room for growth in our understanding of public perspectives on sexual expression and dementia, and its impact on the development of a resident‐ or consumer‐centred policy. Policy discussions have often centred on accreditation and legal standards as well as nursing home rules and regulations;^5^ however, they have lost the consumer voice that is imperative to a truly resident‐centred approach to care. As nursing home care moves towards resident‐ or person‐centred approaches to care,^17^ our policies must reflect that. This includes policies on sexual expression in a nursing home setting.

#### 1.1.3 Public opinion and policy development

Research has shown that public opinion can have a significant effect on policy, with several examples of policy advancement driven by the public voice.^18^, ^19^ This includes key nursing home policy shifts, such as the 1987 Nursing Home Reform Act, thanks to advocacy organizations representing the public voice.^20^ Further, the public has a vested interest in nursing home policies, as 42% of adults over 65 will spend time in a nursing home during their lifetime,^21^ and an even larger proportion will have a loved one in nursing home care. As recommendations for sexual expression policy are developed by experts in nursing home and dementia care, the opinion of (now and future) residents must be taken into account. This will further ensure that sexual expression policy is relevant to all parties and may help to withstand situations such as the Rayhons.

Yet, there is limited knowledge regarding public opinion on sexual expression and dementia within a nursing home.^22^ In April of 2015, we were provided with a rare view into public opinion as the article, “*Sex and Dementia and a Husband on Trial at Age 78*,” was published in the New York Times (NYT).^23^ In response, the public provided several comments on how the case was handled by the nursing home and in the courts. The purpose of this research was to analyse these comments in order to answer the question: “How does the public view sexual consent in the context of dementia in long‐term care?”

## 2 Methods

### 2.1 Design and materials

Aims of this study were descriptive and exploratory; thus, qualitative methods were utilized. This allowed for depth and breadth of information regarding the public opinion of the necessary conditions for sexual consent among older adults with dementia.

Materials analysed consisted of all publicly available comments (N=1194) made online in response to a NYT article, “*Sex and Dementia and a Husband on Trial at Age 78*” posted on April 13, 2015.^23^ The article outlined the case of Donna and Henry Rayhons, who met and married in their 70s. Donna was later moved into a nursing home due to late‐stage Alzheimer's disease. Henry visited daily and they were known to be physically affectionate and even engage in sexual intercourse. Out of concern for Donna, one of her daughters prompted the development of a care plan indicating Donna no longer had the capacity to provide sexual consent, which was communicated to Henry. Shortly after, Henry reportedly visited Donna and drew the curtains around the bed, and her roommate reported hearing sexual noises. Additionally, a video camera showed Henry putting a pair of Donna's underwear into a laundry basket. A rape kit was administered to Donna, which showed no signs of tearing or vaginal semen. However, Henry was later charged with rape and arrested, which hinged on the question of Donna's ability to provide consent. Readers commented on the online article from 13 April 2015 to 15 April 2015, producing 1194 comments. Readers were able to submit an original comment and/or reply to a comment made prior. Notably, the NYT published a follow‐up article on 17 April 2015, “*A Lively Comment Discussion about Dementia and Sex*” about the comments generated over the two days the original article was open for public comment.^24^

### 2.2 Data analysis

Content analysis—a systematic process to describe written communication—was employed to discover emerging patterns among the publically available data.^25^ This methodology is commonly used to analyse written media and is well suited for the analysis of public opinion.^26^ Four researchers independently reviewed the data utilizing constant comparative strategies and inductively identified codes, categories, and one theme. The process was guided by the research question and each researcher's prior experience with the literature on sexual expression in LTC, thus combining conventional and directed processes within an interpretive paradigm.^25^

In order to establish a coding scheme, four researchers initially examined separate sections of the full set of comments and met to present and discuss emerging coding schemes with the full research team, two additional members serving in an advisory capacity but not involved directly in coding. A series of on‐going discussions were utilized to reconcile differences among coding schemes (eg labelling, additional codes, overlapping codes, categorizing), seeking majority consensus. The four coding researchers then applied the coding scheme to several randomly selected sections of codes for refinement and reliability.^27^ Reliability analyses showed a simple agreement among the four coders at 80%. The final coding scheme was then applied to the full data set by the four coding researchers and code frequencies were calculated.

## 3 Findings

All reader comments (N=1194) on the NYT article were examined in the light of the research question, “How does the public view sexual consent in the context of dementia in long‐term care?” With this question as a guide, the analysis resulted in one overall theme, and several related categories.

In addition to the thematic and categorical analysis, it was of interest whether or not the commenters supported sexual expression in the Rayhons' (or a similar) situation. Several comments demonstrated a clear stance for or against sexual expression for individuals with dementia. Of those, 68.3% were rated as “for” sex for individuals with dementia as compared to 31.7% that were “against.” This demonstrates the overall public opinion about sex and dementia in long‐term care. The following describes nuanced details of the thematic and categorical analysis, illustrating how the public views this issue and what “evidence” they cite as important to making their decision.

### 3.1 Theme: Conditions necessary to be sexual

**Figure 1:**
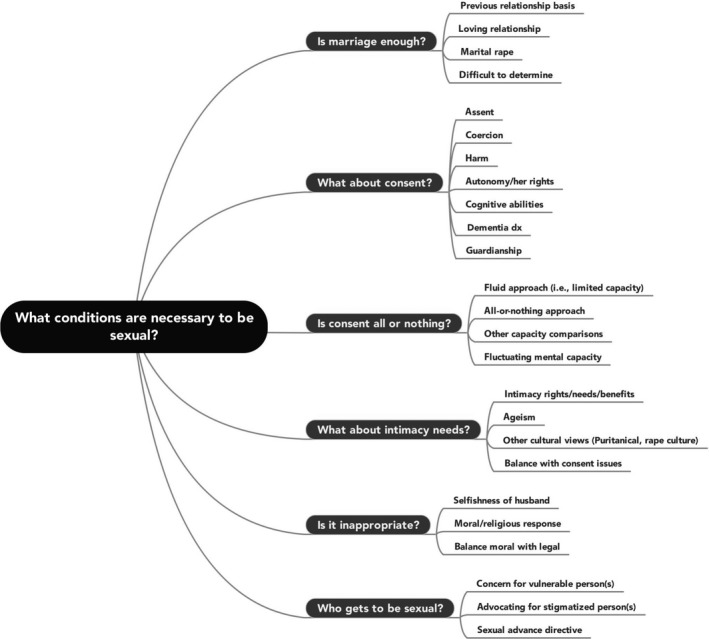
Detailed coding structure for the overall theme

The majority of written comments implied the guilt or innocence of the husband, Mr. Rayhons, based on citing several different conditions necessary for engaging in sexual intercourse when at least one individual has dementia. These “conditions” represented public opinion about sexual consent among older, cognitively compromised individuals. Several codes (N=1348) were assigned to the comments in the overall theme, with six interrelated categories represented (see Figure 1). Although the public identified all of the following conditions as necessary for sexual consent in a situation where one older adult is cognitively compromised, several of the conditions mentioned did not align with commonly accepted legal and clinical standards for sexual consent—understanding, reasoning and voluntariness/assent—at least in the majority of U.S. jurisdictions.^35^ Thus, two broad types of conditions were labelled by the research team and were represented across all categories: (i) *non‐consent* conditions (eg marriage, love, intimacy needs) and (ii) *consent‐related* conditions (eg cognition, harm, assent). Each of the two conditions had similar rates of frequency within the overall theme, with *non‐consent* conditions representing 52.45% (n=707) of the codes and *consent‐related* conditions representing 47.55% (n=641). Commenters would often cite several *non‐consent‐* and *consent‐related* conditions concurrently to argue which conditions are necessary to be sexual when an individual has dementia. Below is a description of key patterns identified within each category.

#### 3.1.1 Category 1: Is marriage enough?

Marriage was a *non‐consent* condition cited as important for an individual with dementia to be sexual. In fact, the central discussion in the overall theme tended to revolve around questioning marriage as a sufficient condition to be sexual—both for and against—with the marriage codes most frequently used within the overall theme (24.41%, n=329). There were several marriage‐is‐sufficient comments, such as:

I cannot believe a HUSBAND is charged with raping his WIFE who has dementia on the THEORY that she could not consent. Goodness, is sex between a husband and wife a crime?

Commenters often cited qualifying conditions to the marriage‐as‐sufficient argument, including a loving relationship, long‐term/established relationship and no history of abuse within the relationship.

If you are in a loving, marital relationship and sex has been welcomed prior to the onset of dementia, the presumption should be that it is welcome even when suffering from dementia.

Some commenters objected, proclaiming marriage is not sufficient and this situation constitutes rape in the absence of evidence the wife could legally consent. Several of the reply streams—several replies to an original comment—evidenced this “marriage vs marital rape” debate, often beginning with a proclamation about the insufficiency of marriage as a sole condition and followed by subsequent arguments. For example, the longest reply stream (59 comments) began with a comment stating this situation was marital rape: “The marriage makes no difference. Sex without consent within a marriage is rape, and has been since the reform of U.S. rape laws decades ago.” Of note, the majority of the replies in that stream were disagreements with this statement, citing *consent‐related* (eg assent, lack of harm) and *non‐consent* conditions (eg loving relationship, intimacy needs) for supporting the sex acts between the Rayhons.

#### 3.1.2 Category 2: What about consent?

The public was also concerned about consenting to sex and the conditions by which consent could be met. To illustrate, a common pattern was asserting that marriage is not sufficient, but is integral to consider along with *consent‐related* conditions (eg wife's assent). For instance, one commenter emphasizes a typical argument that marriage (*non‐consent* condition) and assent (*consent‐related* condition) are both important by stating,

I have been married for nearly 20 years and have had sex with my husband many, many times. Only very rarely have we verbally agreed to sex prior to engaging in it. The majority of the time, our signals to each other are non verbal, possibly quite subtle at first, becoming more obvious as arousal takes place.

Assent—the ability to demonstrate agreement—was emphasized as a pivotal condition for being sexual if the individual has dementia, ranking as most frequent among *consent‐related* codes (n=122) and third among all codes within the overall theme. The assent condition is exemplified in the following:

Here, however, there is no evidence that Ms. Rayhons said “no,” or, in any way, indicated that she did not consent. In fact, the evidence…showed that she was happy to be with her husband. If there was some indication that she did, in fact, object to his advances, then, it would be a completely different story.

Additional *consent‐related* conditions were frequently cited—often in combination—as conditions to be sexual. These included whether there was harm and/or coercion and emphasis on the wife's rights/autonomy. There was a common pattern of supporting the wife's ability to have sex due to these *consent‐related* conditions:

Sexual expression is a fundamental human right. To deny it to people with dementia or with mental illness is unkind…For us to second‐guess him [Mr. Rayhon], shouldn't we have substantive evidence, evidence that she was forced, injured, or unhappy afterward. No such evidence is mentioned here.

*Consent‐related* conditions were also used to argue the wife's inability to have sex, at times instigating the pattern discussed above, when marital rape was implicated. Cognitive limitations were cited by some as evidence the wife could not be sexual.

It makes no difference if they are married or not. She cannot consent. She scored zero on the assessment of memory and orientation. Marital status is not the issue here, it's ability to consent and ability to say no. Mrs. Rayhon's could do neither. This is rape.

Similarly, the diagnosis of Alzheimer's disease was used as a justification for prohibiting sex, as one commenter stated, “This woman has Alzheimer's for God's sake. It is akin to sex with a mentally [ill] patient.”

Occasionally a commenter would suggest the daughter's guardianship as a *consent‐related* condition. For example, “She cannot consent to what she does not understand. Her daughter had legal custody of her and therefore made the decisions about her. Husband had no right to sexually abuse her.” However, the majority of the guardianship comments questioned the decision to award the daughter guardianship and/or referred to the husband as having “next of kin” decision‐making rights. “Why should the nursing home defer to children but not to the spouse?…I certainly hope my kids won't have a say in whether I have sex with my spouse when I am in mental decline.”

#### 3.1.3 Category 3: Is consent all‐or‐nothing?

Commenters often questioned whether inability to consent should necessarily limit sexual expression. For example, one commenter stated, “Why does the opposition rest on lack of consent? Do you know if she indicated any pleasure or sense of being comforted? Did she show physical signs of abuse? Not according to this article.” This was considered a fluid vs an all‐or‐nothing approach to consent and sexual expression. Different *consent‐related* conditions were used to support fluid arguments. For instance, commenters frequently compared the wife's inability to consent to sex with her other capacities, making the argument that consent is not necessary to engage in other activities (eg. feeding, bathing, medications) and this logic should apply to sex.

Wow. She can't be allowed to have sex with her husband, whom she is happy to see when he visits, because she has “feelings” but lacks “good judgment?” We probably can't determine what she would really want for breakfast, either. Does that mean she shouldn't be allowed to eat?

A more fluid approach to consent was also demonstrated in comments about fluctuating mental capacity. Comments mentioned periodic lucidity as a condition to be sexual. “By the way a person with severe dementia can on occasion have moments of lucidity wherein they can indicate needs or desires with physical gestures.”

*Consent‐related* and *non‐consent* conditions were often utilized in combination to demonstrate a fluid approach to sex and dementia. The following comment is a quintessential example.

What does consent mean in this circumstance? Let's say the wife no longer recognized the husband, but welcomed the physical closeness and release of sexual activity. Should she be barred from any sexual activity even if she finds it obviously pleasurable and is able to sleep better or becomes more relaxed?

Other commenters asserted an all‐or‐nothing approach to consent for the Rayhon's situation, indicating that sex without consent should not occur regardless of *non‐consent* conditions. “If she doesn't have capacity to consent, it was rape, plain and simple.”

#### 3.1.4 Category 4: What about intimacy needs?

A substantial portion of the commenters indicated the need for intimacy (*non‐consent* condition) across the lifespan was a decisive factor, both in the specific situation and in general, as a condition to be sexual when a person has dementia. A common plea is displayed here, “The intimacy supersedes all other things in their consistently ever‐narrowing world of function and experience. They need that touch, those caresses, in their ever‐shrinking world.” This was often used in conjunction with criticisms about the ageist and/or Puritanical views of sexuality that dominate society.

It is disturbing to see the sexism and the ageism of some of the comments here. It is also disturbing that the Puritanical views about sex would deprive an elderly woman of one of the few pleasures she can still enjoy.

Some commenters clarified that intimacy needs (*non‐consent* condition) are important and older adults should be viewed as sexual; however, that did not override the importance of lack of consent (*consent‐related* condition) (eg “I am old and agree that old people have sex but consent is always important.”)

#### 3.1.5 Category 5: Is it inapproriate/appropriate?

Commenters also questioned the inappropriateness/appropriateness or moral quality of the sexual acts (*non‐consent* condition), as opposed to its legality (ie consent). Most frequent among these comments were those declaring this was wrong based on Mr. Rayhons being selfish/inattentive to his wife's needs.

Wow. So here's a woman who's so cognitively impaired that she no longer recalls her daughters' names, but her husband can't manage to keep himself zipped up? It smacks of a patriarchal selfishness originating from the belief that a wife's duties—apparently, no matter what—are to provide sex for her husband.

Inappropriateness/appropriateness comments often inferred a moral or religious aspect, such as, “This is plain and simply not morally right. A nursing home is no place for sexual relations. It is just bad form, especially with a roommate nearby.” In contrast, commenters objected to what they observed as judgments based on moral and/or religious grounds. This was exemplified by one commenter stating, “Sometimes it seems like there's nothing the Midwest loves to hate more than sex—or loves to read about more than people getting punished for liking it.” There was also evidence of a more fluid (vs all‐or‐nothing) approach among some of these comments. One commenter wrote,

The only matter I find disgusting here is that Mr. Rayhons obviously crawled into bed with his wife behind a flimsy curtain in a double room, and sexual ‘noises’ were clearly heard by the roommate…On the other hand, nobody should be tried in court for lack of manners.

#### 3.1.6 Category 6: Who gets to be sexual?

Commenters also gave their opinions as to who has the right to be sexual, using both *non‐consent* and *consent‐related* conditions. There was a notable tension between concern/protection and advocacy within this discussion. Several comments were aimed at protecting vulnerable people (eg older adults, demented individuals, LTC residents) from being coerced into sex.

We as a society have an obligation to protect our vulnerable populations. Based on the details provided, someone with her stage of dementia would lack decision‐making capacity related to her health and sex. Yes, I know it is her husband and they have been married for years and, presumably, they had many years of consensual sex. Yet, she was not the same person she once was.

This was often opposed by those advocating for stigmatized groups (eg older, demented adults) to exercise their rights to sex and intimacy. One commenter captured this sentiment when stating, “People with Alzheimer's remain people and people with the ability to love and care.” Commenters also mentioned specific mechanisms to advocate for themselves and others in similar situations. In fact, in one of the longest reply streams (N=26 replies) commenters generated a solution to protect their own sexual rights in advance of having dementia via preparing an advance directive for sexual expression. However, some commenters pointed out the practical application of sexual advance directives may be difficult in settings where it is novel. This is likely true as these documents are far from the norm and the legal recognition of such a document remains unknown in the United States.^7^

## 4 Discussion

This study was conducted to examine public views and expectations regarding sexual expression and dementia within nursing homes. Public opinion about this issue is largely unknown; yet, at a basic level, these results suggest the issue is both highly important to the public and contentious. In fact, the NYT received so many comments (N=1194) within the first two days that the stream was shut down and a subsequent article was published about those comments.^24^ Given that growing numbers of the public are future consumers of nursing home care and currently represent 1.4 million people in US nursing homes annually,^28^ it is imperative their opinion be considered as health‐care and nursing home policies for sexual expression and consent develop.

Overall, comments show the public grappling with the issue of sex and dementia in nursing homes; however, the majority of commenters were in favour of sexual expression for individuals with dementia (68.3%), indicating the general public wants this supported in long‐term care settings. This majority opinion may be in direct opposition to those “in charge” of practices and policy‐making in nursing homes (eg administrators, accreditors) who have often taken a paternalistic stance towards sexual expression for individuals with dementia, resulting in prohibitive practices in many homes.^1^, ^3^ Those commenters with clear dissenting views (31.7%) are more in line with current nursing home practices and also displayed a firm protective stance towards the vulnerable person (ie Mrs. Rayhons). Considering the current paradigm shift in nursing homes towards resident‐centred practices and policy,^17^ sexual expression policy for residents with or without cognitive limitations will need to represent both the majority “for” and minority “against” with flexible policies that balance resident wishes/autonomy with protection and safety.

Analysis also provided insight into specific arguments the public used to justify their decisions. The comments grouped into patterns of arguments, as described above—marriage, consent and cognitive capacity, intimacy, appropriateness, and protection vs advocacy. One major argument was whether marriage is sufficient grounds or if marriage is irrelevant when cognitive capacity limits a person's ability to give consent. This was a highly contentious issue among the public, which implies it must be considered when developing policy and implementing practices within nursing homes. Also, the marriage‐as‐sufficient argument indicates the importance of educating the public and nursing home administration about the specifics of sexual consent law within relevant legal jurisdictions, particularly as it pertains to marital relationships. Currently there are a few states that have provisos for marriage in sexual consent law, and the Rayhons lived in Iowa, which does allow for sexual expression with a cohabitating spouse without mental capacity.^29^ This affects decision making about sexual expression and dementia at a resident and facility level, as it appears in some states a married couple can engage in sexual expression, regardless of capacity.

Another set of arguments centred on the need to protect vs advocate for individuals who cannot communicate their wishes. A unique result from this argument was the suggestion to create a sexual advance directive in order to advocate for your own rights to engage or not engage in sexual expression, according to your written wishes. The acceptance of this type of document remains unknown within the United States;^7^ however, establishing the legitimacy of sexual advanced directives is an integral step to more resident‐centred policy and practice and requires collaboration between nursing homes, consumers/residents and elder law attorneys.

Perhaps the most interesting finding across the comments was the nuance with which the public approaches this issue. Their arguments demonstrated depth of analysis along with impassioned emotional appeals. Although at times the public opinion demonstrated either a *non‐consent* or *consent‐related* opinion, the majority of the comments employed both types of “evidence” within an argument. The public frequently utilized a combination of *consent‐*related and *non‐consent* evidence within each argument type, even commenters arguing about the indecency of the act (*non‐consent*) sometimes conceded that indecent does not equate to illegal (*consent*). This was also exemplified when commenters argued between marriage vs marital rape. The majority of the comments did not solely rely on marriage as insufficient/sufficient evidence (*non‐consent*), but also implicated one or more consent‐related issues such as lack of harm to the wife or observation of her assent (eg enjoyed, welcomed the activity). This suggests a complex decision‐making process is occurring for the general public as they considering sex and dementia, which was illustrated in fluid vs all‐or‐nothing arguments about consent.

The public will expect this issue to be addressed by policies and practices that display a similar level of nuance. In terms of consent law and policy, this is demonstrated best in a limited capacity approach, which suggests that consent is not a black‐and‐white issue but a balance between multiple factors.^30^, ^31^ Considerations for limited capacity include several issues the public raised, such as the level of risk in the proposed intimate activities, potential to mitigate that risk with external support, relationship history/status, capacity of the individual, individual values/wishes and benefits to the individual.^6^ In limited capacity approaches, these factors are all balanced to assess whether the intimate relationship can be supported, regardless of cognitive capacity. Thus, limited capacity provides the flexibility the public wants, and accounts for complex situations that arise within long‐term care, such as the Rayhon's.

### 4.1 Limitations

The analysed comments were limited to NYT readership and may not be representative of the general US population, or other nations. The NYT's article likely attracted comments from a selection of the general readership, which according to the Pew Research Center^32^ is comprised of slightly more men (56%) than women (44%), somewhat younger (32% ages 18‐29, 31% ages 30‐49, 21% ages 50‐64, 12% 65+), educated (56% with college degree, 24% some college, 18% high school or less) and more moderate (35%) to liberal (36%) ideologies. In terms of generalizability of these results, the readership of the NYTs is more likely to be male, somewhat younger and more highly educated than the general US population. This may be similar in other Western/industrialized countries with similar populations to the United States.^33^ However, the breadth and depth of the comment streams represent a range of sentiments from commenters across each region of the United States, and a small number of international readers.

## 5 Conclusions and Implications

Health‐care policy has failed the Rayhons and the public when it comes to how sexual expression is handled within a nursing home setting. Policy related to sexual expression and dementia in nursing homes is scant, and public opinion is largely unknown, as we have spent most of our time in health care attempting to sweep the issue under the proverbial rug. Why? Because it is often contentious, messy, and our approaches are reactive and often fail to take into account the residents' wishes and rights.^1^ In short, policy is inadequate and ill‐informed from both health services and consumer point of view. One solution is to develop informed, resident‐centred policies that provide guidelines to those involved in making these difficult decisions.^3^

The findings are directly relevant to the development of health‐care policy and public policy within the United States, and other countries with limitations in sexual expression policy. What does the public expect in terms of sexual expression policies and procedures? The previous lack of data and subsequent published research in this area left this question unanswerable. However, the Rayhon's case provided a unique opportunity to capture public opinion of sexual expression and dementia within nursing homes on a national scale, providing key information to inform a resident‐centred policy. These findings allow us to begin to understand residents' wishes about sexual expression within nursing home care, and the United States and other countries should continue to seek public opinion data to inform care.

The majority of the public supports sexual expression for individuals with dementia that occurs in a nursing home setting. Their nuanced approach reveals a decision‐making process similar to limited capacity, taking into account several types of evidence with the goal of supporting intimacy and safety, which is in line with recommended practices for sexual consent assessment.^6^, ^7^, ^16^, ^31^ Considering this, policymakers and facility administrators should be examining indicators, such as a loving, caring relationship, the importance of intimacy needs, and the impact of ageism/paternalism along with cognitive capacity, risk and safety. This can be successfully done within a nursing home, as seen with the Hebrew Home at Riverdale.^34^ Taking this flexible, recommended approach can facilitate the development of policies and procedures that capture the resident/consumer voice, protect against harm and support safe sexual expression for individuals living in nursing homes.

## Conflicts of Interest

No conflicts of interest to report for any of the authors.
